# Structural Equation Modelling Reveals That Nutrients and Physicochemistry Act Additively on the Dynamics of a Microcosm-Based Biotic Community

**DOI:** 10.3390/biology8040087

**Published:** 2019-11-14

**Authors:** David A. Russo, Andrew Ferguson, Andrew P. Beckerman, Jagroop Pandhal

**Affiliations:** 1 Department of Chemical and Biological Engineering, University of Sheffield, Mappin Street, Sheffield S1 3JD, UK; andrew.ferguson@sheffield.ac.uk; 2 Department of Animal and Plant Sciences, University of Sheffield, Alfred Denny Building, Western Bank, Sheffield S10 2TN, UK; a.beckerman@sheffield.ac.uk

**Keywords:** algal-bacterial interactions, ecosystem function, eutrophication, microcosm, structural equation modelling

## Abstract

Anthropogenic eutrophication has caused widespread environmental problems in freshwater lakes, reducing biodiversity and disrupting the classic pelagic food chain. Increasing our understanding of the exact role of nutrients and physicochemical variables on microbial dynamics, and subsequent microalgal and cyanobacterial blooms, has involved numerous studies ranging from replicate microcosm-based studies through to temporal studies of real lake data. In a previous experimental microcosm study, we utilised metaproteomics to investigate the functional changes of a microalgal-bacterial community under oligotrophic and eutrophic nutrient levels. Here, we analyse the time series data from this experiment with a combination of typically used univariate analyses and a more modern multivariate approach, structural equation modelling. Our aim was to test, using these modern methods, whether physicochemical variables and nutrient dynamics acted additively, synergistically, or antagonistically on the specific biotic community used in the microcosms. We found that nutrients (nitrogen and phosphorus) and temperature acted additively on the interactions between the microalgae and bacteria present, with the temperature effects elevated in the eutrophic conditions we applied. The data suggests that there may be no synergistic interaction between nutrients and temperature in the tested microcosms. Our approach demonstrates how the application of multivariate methods to existing datasets, in our case from nutrient-enriched freshwater microcosms, enables new information to be extracted, enhancing interpretations as well as allowing more reliable comparisons to similar published studies.

## 1. Introduction

Nutrient enrichment, referred to as eutrophication, can lead to blooms of eukaryotic microalgae or cyanobacteria and associated shifts in the number of trophic levels supported and the diversity of species in lakes and ponds [1]. Understanding the effect of eutrophication on freshwater ecosystems requires an analysis of factors both internal and external to the system, as well as the complex interactions among them. Research into the process of eutrophication is typically centred on a univariate assessment of a subset of indicator variables, including nutrient concentrations, physicochemical factors, and biological characteristics. These studies have regularly identified increasing nutrient concentrations as a key factor responsible for promoting blooms [2,3]. However, more recent studies indicate that physicochemical factors, such as temperature, are also a key factor in microalgal bloom promotion [4,5,6]. Thus, there is no consensus regarding the relative importance of nutrient concentrations and physicochemical factors in bloom promotion.

A feature of this historical research is the use of statistical tools such as correlation and multiple linear regression. For example, Muylaert et al. (2000) used multiple regression to study variation of the phytoplankton community in a freshwater tidal estuary. Phytoplankton dynamics were shown to be controlled by varying irradiance, temperature, and salinity. However more than 40% of species variation remained unexplained [7]. Rigosi et al. (2014), in an analysis of over 1000 USA lakes, used correlations and multiple linear regression to assess how cyanobacterial biovolume and chlorophyll *a* responded to nutrient availability and temperature [8]. Although a full interpretation of the dataset was not possible, the results suggested that in most cases the interaction between nutrient and temperature was additive rather than synergistic. In a later study, Bhattacharya and Osburn (2017) utilised an innovative mix of multiple regression and fluorescence spectroscopy to predict phytoplankton dynamics in a freshwater river network. While it was shown to be a fast and cost-effective method to study large river ecosystems, the models failed to explain more than 50% of the variance in the data [9].

These are just a few examples that illustrate shortcomings of traditional analyses. These models often lack explanatory power, cannot separate correlation from causation, and may reveal an incomplete picture because they fail to capture potential interactions among nutrients, physicochemistry, and the biotic community. Therefore, a multivariate tool with multiple predictor and response variables that captures simultaneous covariation among variables is needed. Structural equation modelling (SEM) can provide such a tool. These have the added advantage of being applicable to existing time-series or spatially resolved data sets, whether these are relatively small microcosms studies or aquatic samples taken from lakes from different sites over many years.

Path analysis is a widely used technique for proposing and testing plausible sets of causal relations among three or more observed variables. As a multivariate technique, it deals explicitly with multiple testing and calculates partial correlations between all variables, controlling for all others [10]. Path analysis does not intend to discover causes but to determine the feasibility of a series of informed hypotheses (i.e., causal paths) based on pre-existing knowledge of the system. By aiming to explain, and not predict, these model structures can avoid the correlation/causation fallacy [11]. 

Traditional path analysis models are somewhat restrictive due to their reliance on single indicators (e.g., NO_3_^−^). SEM improves upon this by allowing the incorporation of multivariate indicator variables (i.e., latent variables). Latent variables refer to variables that cannot be directly inferred by single indicators (i.e., nutrients) and require multiple indicators to capture their essence. Latent variables also provide the opportunity to statistically test ideas about how groups of variables might co-vary together and structure relationships among variables and function. Given the importance and complexity of phytoplankton dynamics, SEM has become an increasingly popular tool to study these phenomena. For example, in recent years, SEM has been successfully used to determine the drivers of diatom diazotroph associations in western tropical north Atlantic blooms [12], test hypotheses regarding the impact of climate factors on algal assemblages in a shallow temperate estuary [13], and explore the biotic and abiotic variables that contributed to the establishment of *Ceratium furcoides* in a shallow eutrophic reservoir [14].

In this study, we utilise SEM, in a proof-of-concept, to explain the dynamics between physicochemical variables, nutrient dynamics, and a microbial community in specific freshwater microcosms. In a previous study, the authors designed freshwater microcosm experiments and applied metaproteomics to investigate the functional changes to algal and bacterial communities, over time, in oligotrophic and eutrophic conditions [15]. Here we apply SEM to the time series from Russo et al. (2016), along with more traditional univariate correlation analyses used in the past. Our aim was to determine the relative importance of bottom-up (NO_3_^−^, PO_4_^3−^, and NH_4_^+^) and physicochemical controls (temperature, dissolved oxygen (DO), and pH) in driving the dynamics of freshwater microbial groups (eukaryotic microalgae, cyanobacteria, and bacteria) under low (oligotrophic) and high (eutrophic) nutrient treatments. 

**Figure 1:**
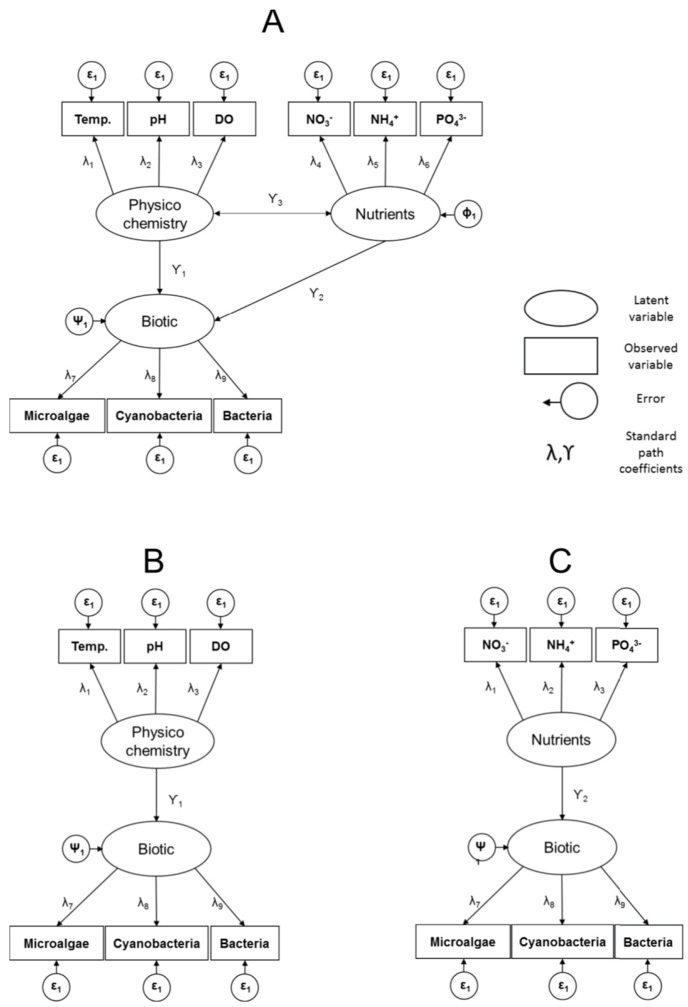
Structural equation models showing proposed relationships between latent variables physicochemistry, nutrients, and biotic. Rectangles represent directly measured variables (e.g., DO). Ovals represent latent variables (e.g., biotic). In model (**A**) a full ecosystem model, incorporating all measured variables, is proposed. Models (**B**,**C**) were proposed to test the effect of the strength and importance of the removed latent variables.

We formulate the structural equation model with a hypothesis that three multivariate, “latent”, variables are central to disentangling the importance of bottom-up vs. physiochemical processes: Nutrients, Physicochemistry and Biotic Interactions (Figure 1A). This path-analytic framework allows evaluation of various hypotheses about the presence and absence of interactions among latent variables, potential direct and indirect interactions among them, and the variance, covariance, and correlation among components of the latent variables.

This study specifically answered the following questions which were not evaluated in the previous metaproteomics study: (1) How do the univariate relationships found in the experimental microbial system compare to previous studies? (2) Do nutrients and physicochemistry act additively, synergistically, or antagonistically to influence the biological component of the microbial community? and (3) Does the nature of the relationships among nutrients, physicochemistry, and biological components of the system vary with nutrient enrichment?

## 2. Materials and Methods

### 2.1. Experimental Design and Sampling

This study is a reanalysis of a previously published time series dataset [15], therefore, only a brief explanation of the experimental design and sampling regime is provided. Briefly, 30 L were housed in controlled environment facilities at the Arthur Willis Environmental Centre at the University of Sheffield, U.K. and filled with 15 L of oligotrophic artificial freshwater growth medium (for detailed composition see Table A1). These were kept under 100 µmol m^−2^ s^−1^ with a 12:12 light dark cycle and no mixing. A microbial community sourced from water samples collected at Weston Park Lake, Sheffield (53°22′56.849″ N, 1°29′21.235″ W) was filtered with a 200 micron fine mesh cloth and utilised to inoculate the microcosms. This was done to remove all non-microbial grazers. The filtered sample was cultured for five days in the conditions described to allow acclimation to the controlled conditions. Subsequently, each 15 L media was inoculated with 2.5 L of this sample. Two nutrient treatments were applied to the microcosms: (1) non-enriched growth medium to simulate oligotrophic conditions (NO_3_^−^ = 0.42 mg L^−1^ and PO_4_^3−^ = 0.03 mg L^−1^) and (2) NO_3_^−^ and PO_4_^3−^-enriched growth medium (NO_3_^−^ = 4.20 mg L^−1^ and PO_4_^3−^ = 0.31 mg L^−1^) to simulate eutrophic conditions. The nutrients were added as NH_4_Cl, KH_2_PO_4_, and K_2_PO_4_ (Appendix A) and levels chosen were based on the oligotrophic and eutrophic ranges according to several international freshwater lake standards [15]. Over the course of the experiment DO, pH, and temperature were measured at 12:00 and 18:00 daily with a Professional Plus Quatro (YSI, Yellow Springs, OH, USA). For the daily estimation of NO_3_^−^, PO_4_^3−^, and NH_4_^+^, 15 mL aliquots were collected, filtered (0.45 µm), and stored until measurement. NO_3_^−^ and NH_4_^+^ were measured with a Dionex ICS-3000 ion chromatograph (Thermo Fisher Scientific, Sunnyvale, CA, USA). PO_4_^3−^ concentrations were estimated according to protocols defined by the International Standards Organization (ISO 6878:2004) [16]. Chlorophyll *a* and phycocyanin fluorescence were measured daily with the AlgaeTorch (bbe Moldaenke GmbH, Schwentinental, Germany). Total heterotrophic bacteria were measured using culturable heterotrophic bacteria as a proxy [17]. Aliquots of 100 µL were plated, in triplicate, on R2A agar (Oxoid, Basingstoke, UK) and incubated for 24 h at 38 °C. Colony forming units (CFU mL^−1^) were counted using OpenCFU software [18].

### 2.2. Pairwise Correlations among Variables

In order to compare the data to previous published studies, where pairwise relationships were made, the Pearson correlation coefficients among all of the variables were estimated. *p*-values were adjusted with a Bonferroni correction to account for multiple testing and, after correction, differences were deemed significant for *p* < 0.001.

### 2.3. Quantifying Direct and Indirect Effects among Functional Biology, Nutrients, and Physicochemistry

SEM was employed to formally test the hypothesis that the latent variables of nutrients and physicochemistry act additively on the biotic community latent variable. The effort simultaneously estimates the strength and direction of covariation among the latent variables, and the covariation among component variables. First, a baseline causal model that allows relationships among nutrients, physicochemistry, and the biotic community during the process of eutrophication was constructed. The baseline model (Figure 1A) is comprised of three latent variables. The physicochemical latent variable is comprised of pH, temperature, and DO. The nutrient latent variable is comprised of NH_4_^+^, NO_3_^−^, and PO_4_^3−^. The biotic latent variable is comprised of colony forming bacterial units (CFU mL^−1^), and microalgal and cyanobacterial concentrations (μg L^−1^). The baseline model allowed the estimation of direct and indirect effects among all latent variables. It also allowed the estimation of variance and covariance among contributing variables within the latent variables (Figure 1A). Two additional models were also specified to evaluate, against Figure 1A, the effect of each of the nutrients and physicochemistry latent variables on the biotic community. The models removed either the latent variable nutrients or physicochemistry (Figure 1B,C). Comparing each reduced model to the full model (Figure 1A) tests the hypotheses regarding the strength and importance of the removed latent variable. The structural equation models were fit in the R Statistical Programming Environment [19] by employing the package “lavaan” [20]; all variables were scaled to one standard deviation prior to analysis.

## 3. Results

**Figure A1:**
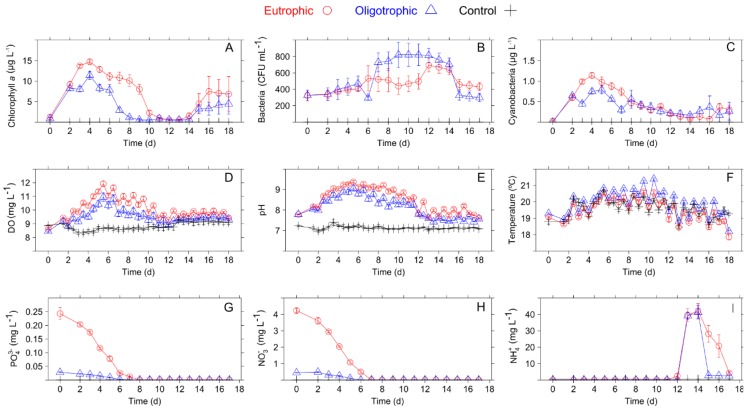
Time series of the measured variables in the oligotrophic and eutrophic treatments. (**A**) microalgae, (**B**) cyanobacteria, (**C**) culturable heterotrophic bacteria, (**D**) DO, (**E**) pH, (**F**) temperature, (**G**) NO_3_^−^, (**H**) PO_4_^3−^, and (**I**) NH_4_^+^ concentrations. Legend is shown in the top left panel (triangles indicate the oligotrophic treatment, circles indicate the eutrophic treatment, and crosses indicate the control). Error bars show standard errors (n = 3). Data adapted from [15].

This study used time series data from Russo et al. (2016) [15]. To support the application of SEM to these data we report them in Appendix A (Figure A1).

### 3.1. Pairwise Correlations among Variables

As in more traditional studies, we made Pearson correlations among variables (we note that cross−correlation analysis is more appropriate for time-series data, but this is rarely used in previous work) [21]. A total of 12 of the 54 pairwise correlations, between physicochemical, nutrient, and biotic variables (Table 1), were significant, after Bonferroni correction, at *p* < 0.001. In the oligotrophic treatment, heterotrophic bacterial concentrations have negative correlations with PO_4_^3−^ (r = −0.44) and microalgal (r = −0.45) concentrations. In the eutrophic treatment, heterotrophic bacterial concentrations have negative correlations with NO_3_^−^ (r = −0.46) and PO_4_^3−^ (r = −0.45) concentrations. Microalgal concentrations have positive correlations, in both the oligotrophic and eutrophic treatments, with DO (r = 0.56 and 0.49, respectively) and pH (r = 0.58 and 0.55, respectively). In addition, microalgal concentrations have a positive correlation with cyanobacterial concentrations in the eutrophic treatment (r = 0.61). Microalgal concentrations have no significant correlations with NH_4_^+^, NO_3_^−^, or PO_4_^3−^ concentrations in either treatments. Cyanobacterial concentrations have a positive correlation with DO (r = 0.65), pH (r = 0.74), and temperature (r = 0.50) in the eutrophic treatment. Cyanobacterial concentrations have no significant correlations with NH_4_^+^, NO_3_^−^, or PO_4_^3−^ concentrations in either treatment.

### 3.2. SEM Analysis: Quantifying Direct and Indirect Effects among Latent Variables Biotic, Nutrients, and Physicochemistry

In the full SEM model, in both the oligotrophic and eutrophic treatments, the partial correlation between physicochemistry and nutrients was not significant (*p* = 0.654 and *p* = 0.987, respectively), indicating independent and thus additive effects of these two variables upon the biological variables and an absence of any indirect effects.

**Figure 2:**
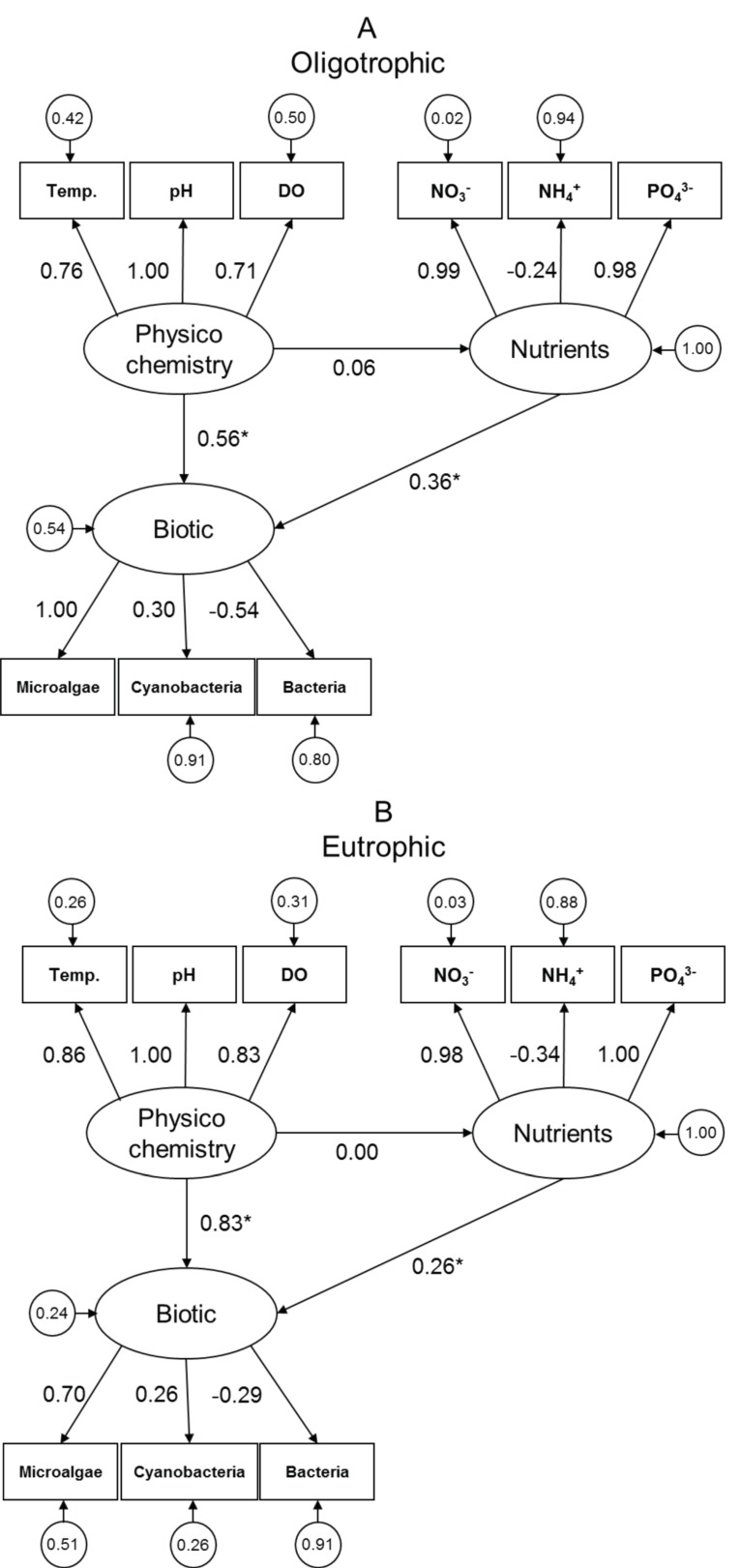
Structural equation models showing proposed relationships between latent variables physicochemistry, nutrients and biotic: (**A**) oligotrophic and (**B**) eutrophic treatments. The numbers in circles correspond to errors. All other numbers correspond to the standardised path coefficients. The asterisks indicate significant values (*p* < 0.05).

Comparing the full model to each of the reduced models, each omitting one of the latent variables, indicated that both nutrients and physicochemistry explained a significant component of variation in microbial dynamics in the experimental aquatic system; this was true for both the oligotrophic and eutrophic treatments (Table 2). Several patterns emerge from the full model comprising inter-relationships among biotic, nutrients, and physicochemistry (Figure 2; asterisks indicate significant values). In both the oligotrophic and eutrophic treatments, the latent variable biotic is more strongly influenced by physicochemistry than by nutrients and the effect of physicochemistry increases in strength with eutrophication (ρ_xy_ (partial correlation coefficient) = 0.56 (*p* = 0.046) in the oligotrophic treatment; ρ_xy_ = 0.83 (*p* < 0.001) in the eutrophic treatment). In contrast, the influence of the nutrient variables decreases from ρ_xy_ = 0.36 (*p* = 0.063) in the oligotrophic treatment to ρ_xy_ = 0.26 (*p* = 0.009) in the eutrophic treatment.

## 4. Discussion

This study aimed to determine the relative importance of nutrient concentrations and physicochemical factors in explaining the variation observed in bacterial, microalgal, and cyanobacterial concentrations over time under oligotrophic and eutrophic conditions, in our specific microcosms. Presently, it is not clear how the interconnectivity of abiotic factors is affected by nutrient enrichment and how nutrients and physicochemistry combine to drive ecosystem productivity [22]. Given that changes in microalgal and cyanobacterial concentrations are the dominant indicator of eutrophication, such an understanding is important for managing freshwater resources. While the results discussed here are specific to the microcosm design and the composition of the microbial community, our analysis demonstrates how the methodology and findings might be extrapolated to natural systems and larger experiments.

Overall, nutrient concentrations and physicochemical factors were found to act additively on the experimental biotic community of microalgae, cyanobacteria, and heterotrophic bacteria, and physicochemical factors exerted a strong influence on both microalgal and cyanobacterial concentrations, which became stronger in eutrophic conditions (Figure 2). In the following sections we scrutinise these observations their ecological context.

### 4.1. Comparison of Observed Univariate Relationships with Previous Studies

Prior to the SEM analysis, one of the objectives was to assess whether the abstracted experimental system still generated commonly found correlations among variables, whether or not these are correct and capable of providing inference. For example, previous research indicates that microalgae and cyanobacterial concentrations are expected to have a strong positive correlation with NO_3_^−^ and PO_4_^3−^ [2]. General consensus is also that the magnitude and duration of the blooms increase with increasing nutrient loads [2]. This provides an immediate assessment of how our microcosms compared under nutrient enrichment. In our pairwise analysis of both experimental conditions (Table 1), NO_3_^−^ and PO_4_^3−^ concentrations did not have a significant correlation with microalgal or cyanobacterial concentrations in either of our treatments. However, two phases of exponential growth were observed in the variation of microalgal and cyanobacterial concentrations. The latter exponential growth phase coincides with the appearance of detectable NH_4_^+^ concentrations (Figure A1I) following the peak of bacterial abundance. This sudden increase in NH_4_^+^ concentrations may be due to bacterial mineralization, possibly promoted by an increase in organic nitrogen in the media following microbial cell lysis. Overall, the lack of correlation between NO_3_^−^/PO_4_^3−^ and microalgae/cyanobacteria could be due to the fact that the microcosms in this study lacked the complexity of natural environments where variations in parameters, such as water depth and stratification, and environmental gradients of dissolved organic carbon and minerals can drive the timing of bloom events [23,24,25].

Due to the lack of correlation between phytoplankton and nutrients, we proceeded to assess whether there were any significant correlations between microalgae or cyanobacteria and physicochemical variables. The pairwise analysis (Table 1) showed a significant positive correlation between cyanobacterial abundance and temperature was confirmed in the eutrophic treatment (r = 0.50). It has been shown previously that, under nutrient-enriched conditions, cyanobacterial species are favoured, in detriment of green microalgae, with an increase in water temperature [6,25]. This is also in line with recent limnological studies where a strong correlation between cyanobacterial concentrations and temperature has been observed [26,27]. In freshwater ecosystems, temperature drives cyanobacterial concentrations directly, through increased growth rates, and indirectly, through its influence on hydrological processes.

### 4.2. Utilising SEM to Quantify Direct and Indirect Effects among Functional Biology, Nutrients, and Physicochemistry

In this proof-of-concept study, SEM was successfully used to address a substantial multivariate question: is the biological response to increasing nutrient enrichment an additive function of nutrients and physicochemistry, or does the effect of nutrient dynamics on the biotic community depend on physicochemistry (i.e., is there an interaction between them). In the full model, in both the oligotrophic and eutrophic treatments, the partial correlation between physicochemistry and nutrients was not significant. This suggests that the effects are additive. This contrasts with recent literature suggesting that the interaction between nutrient concentrations and physicochemical factors may be synergistic [28,29]. This opinion may stem from an overrepresentation of studies that focus on eutrophic environments where the interaction between temperature and nutrient is enhanced [8,27,30]. Furthermore, the independent strength and significance of nutrients and physicochemistry was assessed by comparing this additive model to models without one or the other. This assessment, capturing several components of the nutrient dynamics and physicochemical components, revealed that nutrients and physicochemistry were both necessary to describe the dynamics of the biological variables and their response to enrichment.

Finally, in addition to this high-level, multivariate assessment of function, the partial correlation coefficients from the SEM allowed a comparison of specific pairwise variable relationships, which have been used extensively in the past. As noted above, no evidence was found for an interaction between nutrients and physicochemistry. Furthermore, the direct effects (Figure 2) of nutrients (ϒ = 0.36 Oligo; ϒ = 0.26 Eutrophic) were much weaker than physicochemistry (ϒ = 0.56 Oligo; 0.83 Eutrophic). This generally agrees with both our dataset and published work that supports the ongoing hypothesis that, in eutrophic conditions, physicochemistry may become a driver of phytoplankton abundance [8,27,30].

The application of an SEM-based analysis to the multiple, potentially direct and indirect interactions, among several features of aquatic communities, provides a holistic approach to understand ecosystem drivers. SEM allows for the inclusion of multiple dependent variables and biologically meaningful collections of them, i.e., latent variables, to obtain a better overall picture of the system. Additionally, as can be seen in this study, each path represents a potential causal hypothesis based on pre-existing knowledge of the system. Therefore, by explicitly testing strong hypotheses (e.g., changes in nutrient levels cause changes in the biotic component of the system), SEM avoids potential issues such as autocorrelation. However, there are some shortcomings that need to be recognised. First, the obtained datasets are relatively small; however, the fact that the conclusions are supported by previously published work provided confidence in the explanatory power of the model. Second, some of the parameters selected in this study do not act in a strictly unidirectional manner. For example, it is known that both DO and pH will vary as a consequence of variation in community composition (i.e., ratio between autotrophic and heterotrophic production) [22]. In terms of SEM analysis, this bidirectionality has the potential to exacerbate the apparent correlation between the physicochemical and biotic components. Overall, this study showed that although artificial microcosms have the potential to be used as tools to develop novel hypotheses regarding natural phenomena, an SEM approach enables more insightful comparisons using existing data sets. It also highlights directions for further work, including increased data collection and more complex experimental setups (e.g., higher trophic levels and environmental gradients), which are required in order to truly elucidate the network of interactions established in the process of eutrophication.

## 5. Conclusions

This study aimed to demonstrate how SEM can be applied to existing ecology datasets to generate new hypotheses and understanding of direct and indirect interactions between measured parameters. Here, we explored the array of effects between physicochemistry and nutrient concentrations and their influence on the microbial community, which, via productivity and nutrient recycling, define the difference between low and high levels of nutrients (eutrophication) in our experimental system. The results show that the dynamics of the experimental community in this study were weakly correlated to nutrient enrichment and, in eutrophic conditions, physicochemical factors became superior predictors of biological variables. Lastly, in line with recent studies, the model results showed that nutrients and physicochemical factors have an independent but additive effect upon the biotic variables. Despite the results being comparable to previous studies, it is important to note that this study was undertaken in artificial microcosms with a limited timeframe and a reduced number of overly simplistic variables. All these factors can potentially influence the results of the study. Future work will move from artificial to natural enclosures and expand the measured variables to provide a closer approximation of the local environment.

## Figures and Tables

**Table 1:** Correlation matrix of measured variables. The asterisks indicate significant values after Bonferroni correction (*p* < 0.001).

Variable 1	Variable 2	Oligotrophic	Eutrophic
Bacteria	NO_3_^−^	−0.40	−0.46 *
	PO_4_^3−^	−0.44 *	−0.45 *
	NH_4_^+^	0.22	0.36
	Temp.	0.27	−0.03
	DO	−0.08	0.00
	pH	0.02	−0.15
Microalgae	NO_3_^−^	0.38	0.20
	PO_4_^3−^	0.40	0.21
	NH_4_^+^	−0.31	−0.37
	Temp.	0.17	0.29
	DO	0.56 *	0.49 *
	pH	0.58 *	0.55 *
Cyanobacteria	NO_3_^−^	0.06	0.14
	PO_4_^3−^	0.06	0.17
	NH_4_^+^	−0.23	−0.41
	Temp.	0.37	0.50 *
	DO	0.33	0.65 *
	pH	0.41	0.74 *
Temperature	NO_3_^−^	0.08	0.19
	PO_4_^3−^	0.07	0.16
	NH_4_^+^	−0.37	−0.48 *
DO	NO_3_^−^	−0.19	−0.33
	PO_4_^3−^	−0.13	−0.31
	NH_4_^+^	−0.06	−0.22
pH	NO_3_^−^	0.04	0.03
	PO_4_^3−^	0.10	0.00
	NH_4_^+^	−0.36	−0.57 *

**Table 2:** SEM model comparison, reporting outcome of likelihood ratio test between full and reduced models. (**A**) Full ecosystem model. (**B**) Reduced model excluding the latent variable nutrients. (**C**) Reduced model excluding the latent variable water quality.

Model Comparison	Condition	DF Difference	χ^2^ Difference	*p*-Value
A vs. B	Oligotrophic	16	71.558	<0.001
	Eutrophic	16	73.305	<0.001
A vs. C	Oligotrophic	16	46.881	<0.001
	Eutrophic	17	76.334	<0.001

## Appendix A

**Table A1:** Complete composition of artificial freshwater growth medium (mg L^−1^).

NaHCO_3_	192
MnCl_2_·4H_2_O	0.18
MgSO_4_7H_2_O	115
KCl	0.45
H_2_SeO_3_	0.0016
Ca(NO_3_)_2_4H_2_O	0.8
NH_4_Cl	1
KH_2_PO_4_	0.025
K_2_PO_4_	0.025
ZnSO_4_·7H_2_O	0.022
Na_2_EDTA.2H_2_O	0.5
H_3_BO_3_	0.114
FeSO_4_·7H_2_O	0.05
CuSO_4_·5H_2_O	0.016
CoCl_2_·6H_2_O	0.016
(NH_4_)_6_Mo7O_24_·4H_2_O	0.011
